# Adzuki Bean Alleviates Obesity and Insulin Resistance Induced by a High-Fat Diet and Modulates Gut Microbiota in Mice

**DOI:** 10.3390/nu13093240

**Published:** 2021-09-17

**Authors:** Qingyu Zhao, Dianzhi Hou, Yongxia Fu, Yong Xue, Xiao Guan, Qun Shen

**Affiliations:** 1 College of Food Science and Nutritional Engineering, China Agricultural University, Beijing 100083, China; zqy565527877@163.com (Q.Z.); xiaozhihou90@126.com (D.H.); fyx2012314095@163.com (Y.F.); xueyong@cau.edu.cn (Y.X.); 2 National Center of Technology Innovation (Deep Processing of Highland Barley) in Food Industry, Beijing 100083, China; 3 National Engineering Research Center for Fruit and Vegetable Processing, Beijing 100083, China; 4 Key Laboratory of Plant Protein and Grain Processing, Beijing 100083, China; 5 School of Medical Instrument and Food Engineering, University of Shanghai for Science and Technology, Shanghai 200093, China; gnxo@163.com

**Keywords:** adzuki bean, obesity, insulin resistance, gut microbiota, PICRUSt2 analysis

## Abstract

Adzuki bean consumption has many health benefits, but its effects on obesity and regulating gut microbiota imbalances induced by a high-fat diet (HFD) have not been thoroughly studied. Mice were fed a low-fat diet, a HFD, and a HFD supplemented with 15% adzuki bean (HFD-AB) for 12 weeks. Adzuki bean supplementation significantly reduced obesity, lipid accumulation, and serum lipid and lipopolysaccharide (LPS) levels induced by HFD. It also mitigated liver function damage and hepatic steatosis. In particular, adzuki bean supplementation improved glucose homeostasis by increasing insulin sensitivity. In addition, it significantly reversed HFD-induced gut microbiota imbalances. Adzuki bean significantly reduced the ratio of *Firmicutes*/*Bacteroidetes* (*F*/*B*); enriched the occurrence of *Bifidobacterium*, *Prevotellaceae*, *Ruminococcus_1*, *norank_f_Muribaculaceae*, *Alloprevotella*, *Muribaculum*, *Turicibacter*, *Lachnospiraceae_NK4A136_group*, and *Lachnoclostridium*; and returned HFD-dependent taxa (*Desulfovibrionaceae*, *Bilophila*, *Ruminiclostridium_9, Blautia*, and *Ruminiclostridium*) back to normal status. PICRUSt2 analysis showed that the changes in gut microbiota induced by adzuki bean supplementation may be associated with the metabolism of carbohydrates, lipids, sulfur, and cysteine and methionine; and LPS biosynthesis; and valine, leucine, and isoleucine degradation.

## 1. Introduction

At present, public health is threatened by the increasing occurrence of obesity worldwide. It is reported that 20% of the adult population will suffer from obesity by 2030 [1]. Excessive consumption of high-fat diets (HFDs) can easily lead to obesity [2]. The disease is multifactorial, usually accompanied by dyslipidemia, hepatic steatosis, insulin resistance, and gastrointestinal diseases. Current obesity treatment measures include increasing energy expenditure, suppressing appetite, inhibiting fat production, and regulating gut microbiota [3]. A HFD can cause changes in the gut microbiota composition and function [4]. Studies have found that gut microbiota is related to lipid metabolism and energy homeostasis, which implies that gut microbiota is significant in obesity and related complications development [5]. More and more evidence shows that functional food, especially beans, can regulate the composition of gut microbiota, and thereby inhibit metabolic disorders development [6,7].

Among the 12 most important cereal and legume crops, adzuki bean (*Vigna angularis*) is consumed in Asia [8]. It is planted in more than 30 countries and regions, of which China has the largest production [9]. According to the Chinese pharmacopoeia, adzuki bean can be used in the treatment of diuresis, swelling, and abscesses. Since the time of the Tang Dynasty in China, adzuki bean has been used for weight control [10]. In addition, the dietary fiber, polysaccharides, protein, and bioactive substances (e.g., polyphenols and saponins) of adzuki bean were found to have many health benefits, such as anti-diabetes [11], anti-obesity [6,8,10,12], liver protection [13], and antioxidant activity [14]. Although public interest in adzuki bean is increasing, current research mainly focuses on either single components or extracts of adzuki bean. In most cases, adzuki bean is usually consumed in the form of the whole bean. However, the biological benefits of whole adzuki bean seeds are not currently well studied. Previous research has indicated that 30% adzuki bean supplementation can significantly decrease serum glucose, low-density lipoprotein cholesterol (LDL-C), and total cholesterol (TC) levels, and improve the glucose tolerance of mice with diabetes induced by a HFD combined with streptozotocin [15]. However, consumption of such a large amount of adzuki bean in the daily diet would be difficult. Therefore, it is not clear whether whole adzuki bean supplementation has beneficial effects on obesity and its complications induced by HFD, especially in the case of intake of a more practical low dose (15%). Furthermore, the relationship between the health benefits of adzuki bean supplementation and gut microbiota also needs to be clarified.

In order to explore the effects of adzuki bean supplementation to improve obesity and gut microbiota imbalances induced by HFD, changes in histological, physiological, and biochemical parameters, and serum LPS level were measured. The regulatory effect of adzuki bean supplementation on the gut microbiota composition was also studied. This work in a model system will enhance scientific understanding of adzuki bean as a functional food to prevent obesity, including the influence of gut microbiota.

## 2. Methods

### 2.1. Material Preparation

Adzuki bean was purchased from Dongfangliang Life Technology Co., Ltd. (Datong, China), and pulverized into powder (80-mesh). The composition of the adzuki bean powder was measured (Supplementary Table S1).

### 2.2. Animals and Diets

Four-week-old male C57BL/6 mice was purchased from Vital River Laboratory Animal Technology Co., Ltd. (Beijing, China). Mice were housed under controlled conditions (24 ± 2 °C, 12 h light/dark cycle, 55% ± 5% humidity) with free access to food and water. After adaptive feeding for one week, all mice were randomly subdivided into three groups (*n* = 8 per group) that were treated for 12 weeks. Specifically, (1) normal control diet (NCD, 10% energy derived from fat, 3.85 total kcal/g), (2) high-fat diet (HFD, 60% energy derived from fat, 5.24 total kcal/g), and (3) HFD supplemented with 15% adzuki bean flour (HFD-AB, 60% energy derived from fat, 5.24 total kcal/g). Daily intake of 250–400 g of cereal-grain, including 50–150 g of whole grain and pulses, was recommended based on the Dietary Guidelines for Chinese Residents (2016). It suggested a 30% maximum intake level for pulses. The 15% adzuki bean dietary supplementation level used in the present study refers to previous studies [6,13,15]. Detailed diet compositions and energy densities are listed in Supplementary Table S2. The HFD (D12492) and NCD (D12450J) diets were from Research Diets Inc. (New Brunswick, NJ, USA). Body weight and food intake were assessed weekly. Feces were collected, and mice were sacrificed after a 12 h overnight fast at the end of experiment. White adipose tissue (epididymal, retroperitoneal, and perirenal fat) and liver were weighed. 

### 2.3. Biochemical Analysis

The serum TC, triacylglycerol (TG), LDL-C, high-density lipoprotein cholesterol (HDL-C), alanine aminotransferase (ALT), aspartate aminotransferase (AST), and fasting blood glucose were measured with an automatic biochemistry analyzer (Hitachi Ltd., Tokyo, Japan). Fasting serum insulin was detected using commercial mouse insulin ELISA kit (ALPCO, Salem, NH, USA). Serum concentrations of LPS were detected with commercial enzyme-linked immunosorbent assay kit from Shanghai Enzyme-linked Biotechnology Co., Ltd. (Shanghai, China), based on the manufacturer’s protocol. 

### 2.4. Oral Glucose Tolerance Test (OGTT)

After 11 weeks, mice were orally administered 2 g/kg body weight of 25% glucose solution after overnight fasting for 12 h. Blood glucose concentration was measured at 0, 15, 30, 60, 90, and 120 min.

### 2.5. Histological Analysis

Samples of liver and adipose tissue were fixed in 4% paraformaldehyde. Dehydrated tissues were paraffin-embedded, sliced to 5 μm thickness, and stained with hematoxylin and eosin (H&E). In addition, the liver samples were stained with Oil Red O solution.

### 2.6. Gut Microbiota Analysis

Bacterial genome DNA was extracted from fecal samples with the E.Z.N.A.^®^ soil DNA kit. Then, 1% agarose gel was used to check the purity of the DNA extract. The DNA purity and concentration were determined using a NanoDrop 2000 UV–Vis spectrophotometer. The V3-V4 of bacterial 16S rRNA gene was amplified with primer pairs 338F (5′-ACTCCTACGGGAGGCAGCAG-3′) and 806R (5′-GGACTACHVGGGTWTCTAAT-3′) with ABI GeneAmp^®^ 9700 PCR thermocycler. The PCR product was extracted from 2% agarose gel, purified with a AxyPrep DNA Gel Extraction Kit, and quantified with a Quantus™ Fluorometer. The sequences obtained were placed into operational taxonomic units by clustering 97% sequence similarity.

The α diversity analysis was performed using Mothur (version V.1.30.2). Both nonmetric multidimensional scaling (NMDS) and principal coordinate analysis (PCoA) were applied to quantify the compositional differences between the microbial communities. ANOSIM analysis was used to test for significant differences in clusters among the groups. The relative abundances of different bacterial communities were assessed with the Wilcoxon rank-sum test at a confident level of 95%. The bacterial biomarkers within groups were explored by the linear discriminant analysis effect size (LEfSe, LDA > 3). PICRUSt2 version 2.2.0 was used to predict the gut microbial metabolic functions based on the 16S sequences.

### 2.7. Statistical Analysis

Statistical analyses were performed with SPSS 22.0 (SPSS Inc., Chicago, IL, USA). All data were shown as mean ± SEM. Significant group differences were determined by one-way ANOVA followed by Duncan’s test (*p* < 0.05). Graphs were prepared by Prism 5 (La Jolla, CA, USA).

## 3. Results

### 3.1. Adzuki Bean Supplementation Alleviated Obesity Induced by HFD

**Figure 1:**
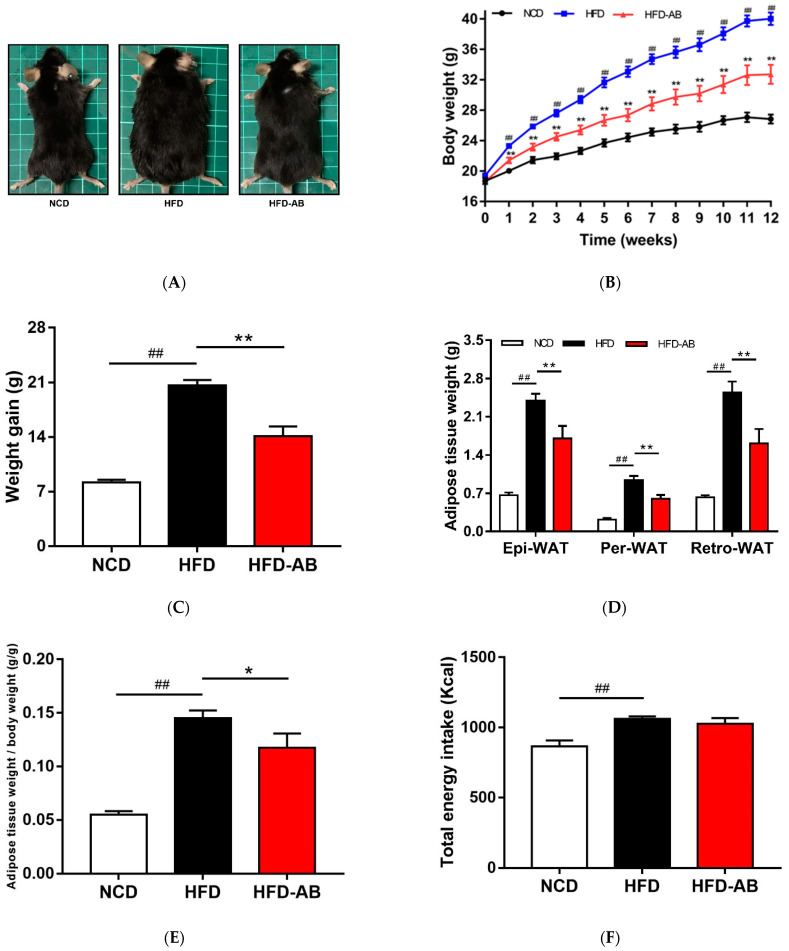

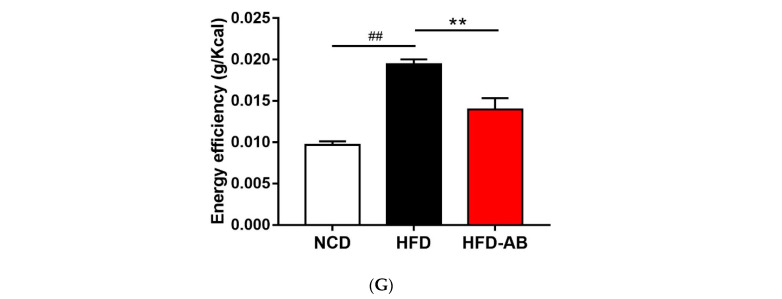
Adzuki bean supplementation prevented HFD-induced obesity. (**A**) Representative pictures of each group taken at the end of the experiment; (**B**) body weight evolution; (**C**) 12-week weight gain; (**D**) the mass of perirenal white adipose tissue (Per-WAT), epididymal white adipose tissue (Epi-WAT), and retroperitoneal white adipose tissue (Retro-WAT); (**E**) body fat ratio; (**F**) total energy intake; and (**G**) energy efficiency. Values are expressed as mean ± SEM, *n* = 8 per group. ## *p* < 0.01, HFD compared with NCD mice. * *p* < 0.05 and ** *p* < 0.01, HFD-AB compared with HFD mice. NCD, normal control diet; HFD, high-fat diet; HFD-AB, high-fat diet supplemented with adzuki bean.

Compared with the NCD group, HFD feeding led to a significant increase in final body weight and weight gain (Figure 1A–C), which was ameliorated by adzuki bean supplementation. The white adipose tissue weight and body fat ratio of the HFD group mice increased significantly (Figure 1D,E). In contrast, adzuki bean treatment significantly reduced the white adipose tissue weight and body fat ratio compared with the HFD group, for which these were consistent with body weight changes. In addition, the energy intake of HFD group mice was significantly higher than that of NCD group mice, but there was no significant difference between the HFD-AB and HFD groups (Figure 1F). The result means that adzuki bean supplementation reduces the abnormal weight gain caused by HFD, and the effect was not the result of reduced energy consumption. The energy efficiency of mice was significantly reduced by supplementation with adzuki bean (Figure 1G), indicating that not all energy was used for weight gain.

### 3.2. Effect of Adzuki Bean Supplementation on Serum Parameters

**Figure 2:**
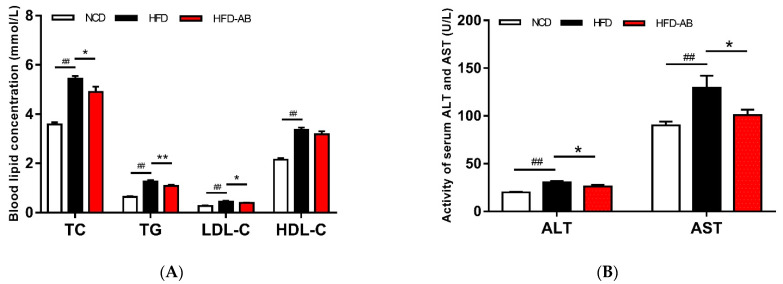

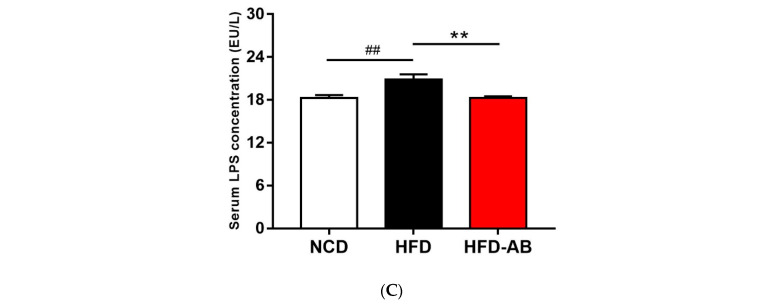
Effects of adzuki bean supplementation on the serum parameters of mice. (**A**) The concentration of triacylglycerol (TG), total cholesterol (TC), high-density lipoprotein cholesterol (HDL-C), and low-density lipoprotein cholesterol (LDL-C); (**B**) serum ALT and AST levels; and (**C**) serum LPS level. Data are shown as mean ± SEM of 7 mice per group. ## *p* < 0.01, HFD compared with NCD mice. * *p* < 0.05 and ** *p* < 0.01, HFD-AB compared with HFD mice. NCD, normal control diet; HFD, high-fat diet; HFD-AB, high-fat diet supplemented with adzuki bean.

Compared with the NCD group, the serum TG, TC, LDL-C, and HDL-C levels of the HFD group mice significantly increased (Figure 2A). In contrast, adzuki bean treatment significantly reduced serum TG, TC, and LDL-C levels compared with the HFD group, but there was no effect on HDL-C level. The levels of AST and ALT of HFD group mice were significantly higher than those in the NCD group (Figure 2B), while adzuki bean supplementation significantly decreased serum ALT and AST. In addition, adzuki bean supplementation significantly reduced the high level of serum LPS (Figure 2C), indicating that adzuki bean can alleviate the metabolic endotoxemia induced by HFD.

### 3.3. Adzuki Bean Supplementation Limited Hepatic Steatosis and Lipid Accumulation

**Figure 3:**
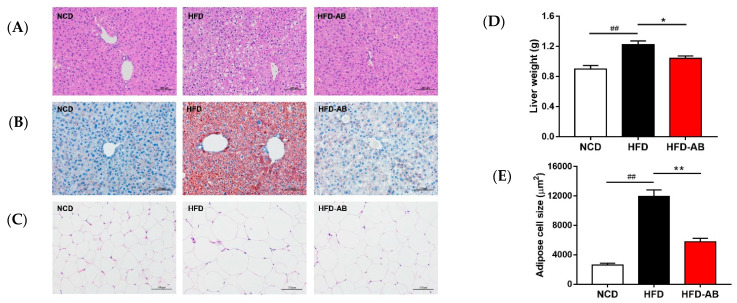
Effects of adzuki bean supplementation on (**A**) H&E staining of liver, (**B**) Oil Red O staining of liver, (**C**) H&E staining of epididymal fat tissue sections, (**D**) liver weight, and (**E**) adipose cell size. Data are shown as mean ± SEM, *n* = 8 per group. ## *p* < 0.01, HFD compared with NCD mice. * *p* < 0.05 and ** *p* < 0.01, HFD-AB compared with HFD mice. NCD, normal control diet; HFD, high-fat diet; HFD-AB, high-fat diet supplemented with adzuki bean.

Histological analysis indicated that adzuki bean supplementation effectively inhibited hepatic steatosis and lipid accumulation (Figure 3A,B). The liver tissue weight and fat-cell size in epididymal fat of the HFD group mice increased significantly compared with the NCD group (Figure 3C–E), and these effects were ameliorated by adzuki bean supplementation. 

### 3.4. Adzuki Bean Supplementation Improved Insulin Resistance Induced by HFD

**Figure 4:**
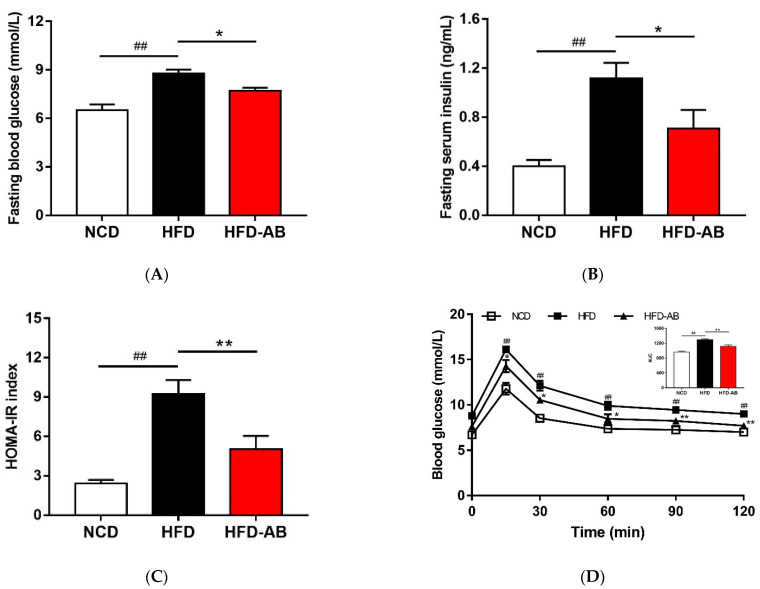
Adzuki bean supplementation improved glucose homeostasis. (**A**) Fasting blood glucose level, (**B**) fasting serum insulin level, (**C**) HOMA-IR index, and (**D**) glucose levels during the oral glucose tolerance test (OGTT). Data are shown as mean ± SEM, *n* = 7–8 per group. ## *p* < 0.01, HFD compared with NCD mice. * *p* < 0.05 and ** *p* < 0.01, HFD-AB compared with HFD mice. NCD, normal control diet; HFD, high-fat diet; HFD-AB, high-fat diet supplemented with adzuki bean.

Compared with the NCD group, HFD group mice had higher serum insulin and glucose levels, and higher HOMA-IR (Figure 4A–C). Conversely, adzuki bean supplementation showed improved serum insulin and glucose levels, and led to a significant decrease in HOMA-IR, indicating that adzuki bean supplementation enhanced insulin sensitivity. The results of OGTT indicated that the AUC of HFD-AB group mice was significantly lower than that of HFD group mice (Figure 4D).

### 3.5. Adzuki Bean Supplementation Regulated Gut Microbiota Dysbiosis

**Figure 5:**
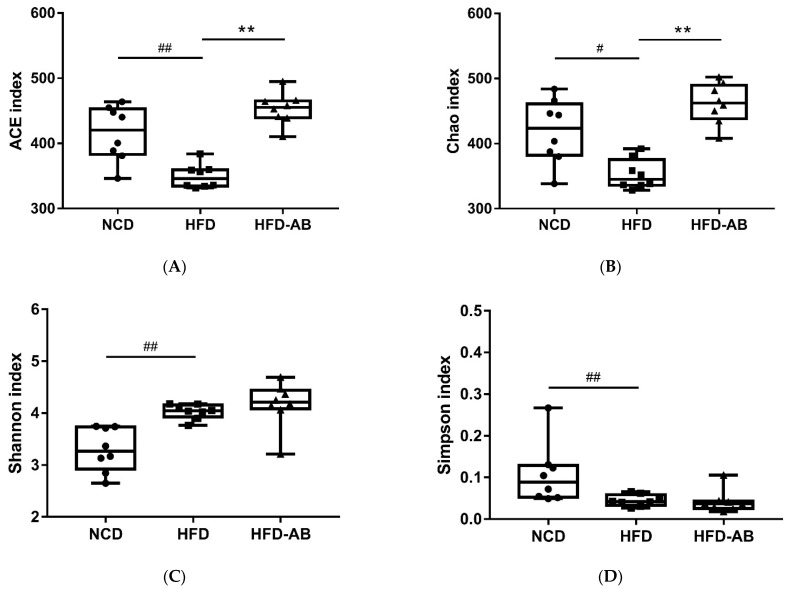

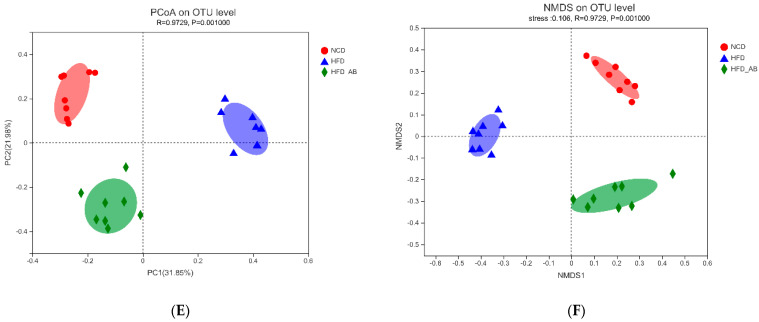
Effects of adzuki bean supplementation on the α and β diversity of gut microbiota in mice (*n* = 8 per group). (**A**,**B**) The community richness accessed by the ACE and Chao indices, (**C**,**D**) the community diversity accessed by the Shannon and Simpson indices, and (**E**,**F**) Principal coordinate analysis (PcoA) score plot and nonmetric multidimensional scaling (NMDS) score plot based on Bray–Curtis. # *p* < 0.05 and ## *p* < 0.01, HFD compared with NCD mice. ** *p* < 0.01, HFD-AB compared with HFD mice. NCD, normal control diet; HFD, high-fat diet; HFD-AB, high-fat diet supplemented with adzuki bean.

**Figure 6:**
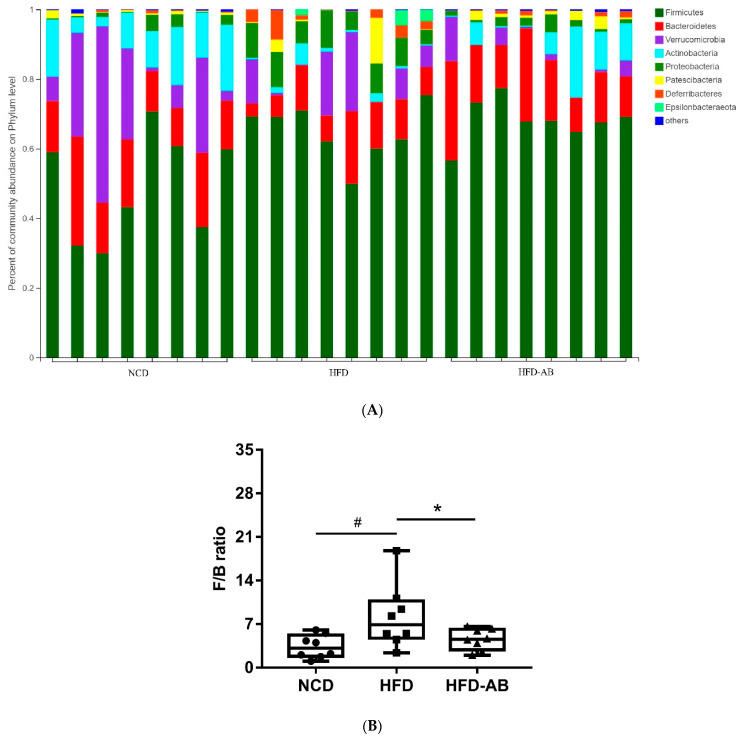

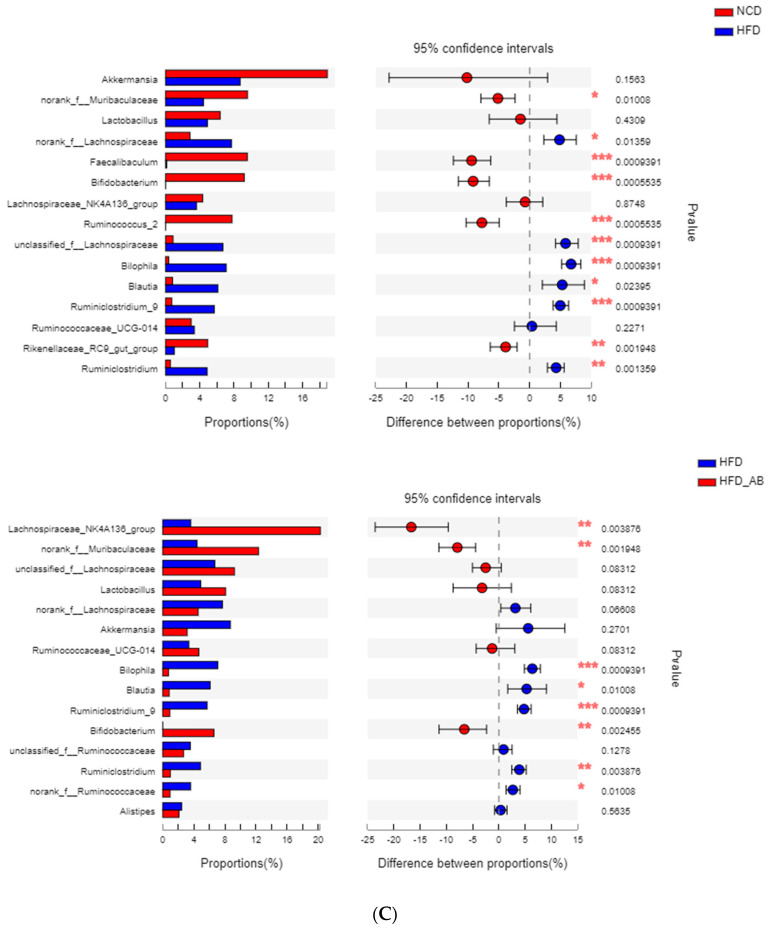
Adzuki bean supplementation modulated gut microbiota composition (*n* = 8 per group). (**A**) The abundances of gut microbiota at the phylum level, (**B**) the *F*/*B* ratio, and (**C**) mean proportions of key genera in different groups. # *p* < 0.05, HFD compared with NCD mice. * *p* < 0.05, HFD-AB compared with HFD mice (in Figure 6B). * *p* < 0.05, ** *p* < 0.01, *** *p* < 0.001 (in Figure 6C). NCD, normal control diet; HFD, high-fat diet; HFD-AB, high-fat diet supplemented with adzuki bean.

**Figure 7:**
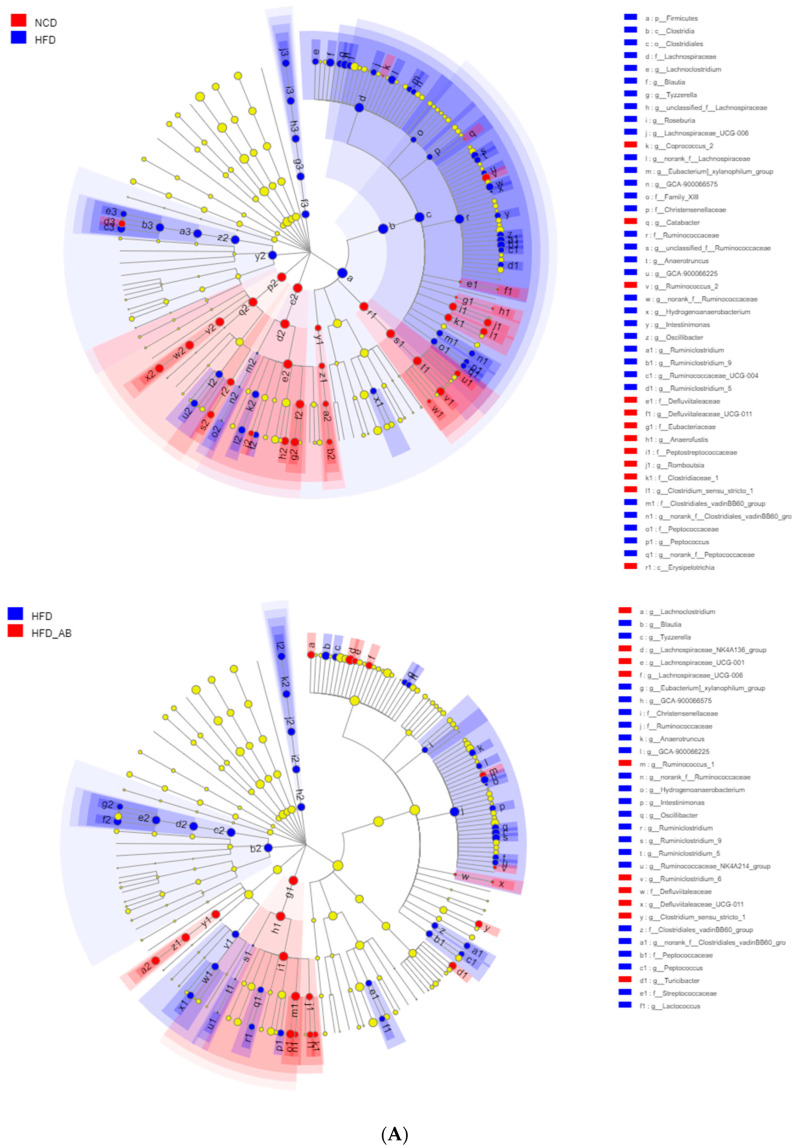

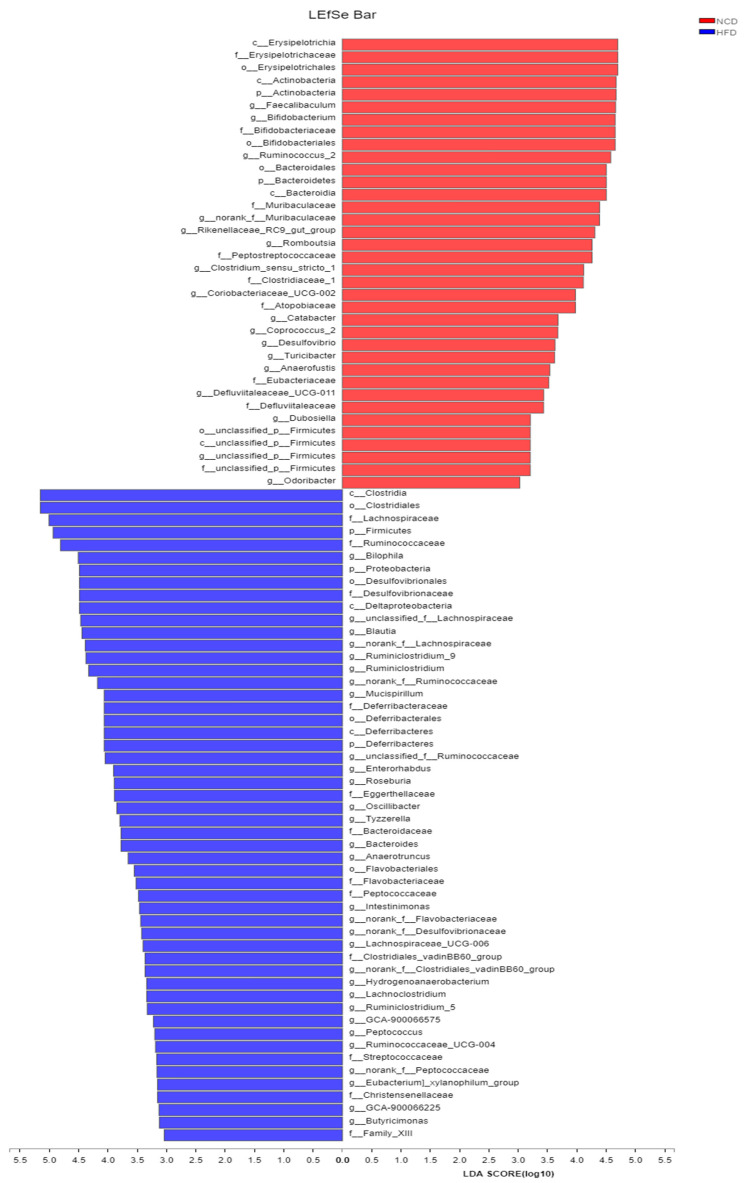

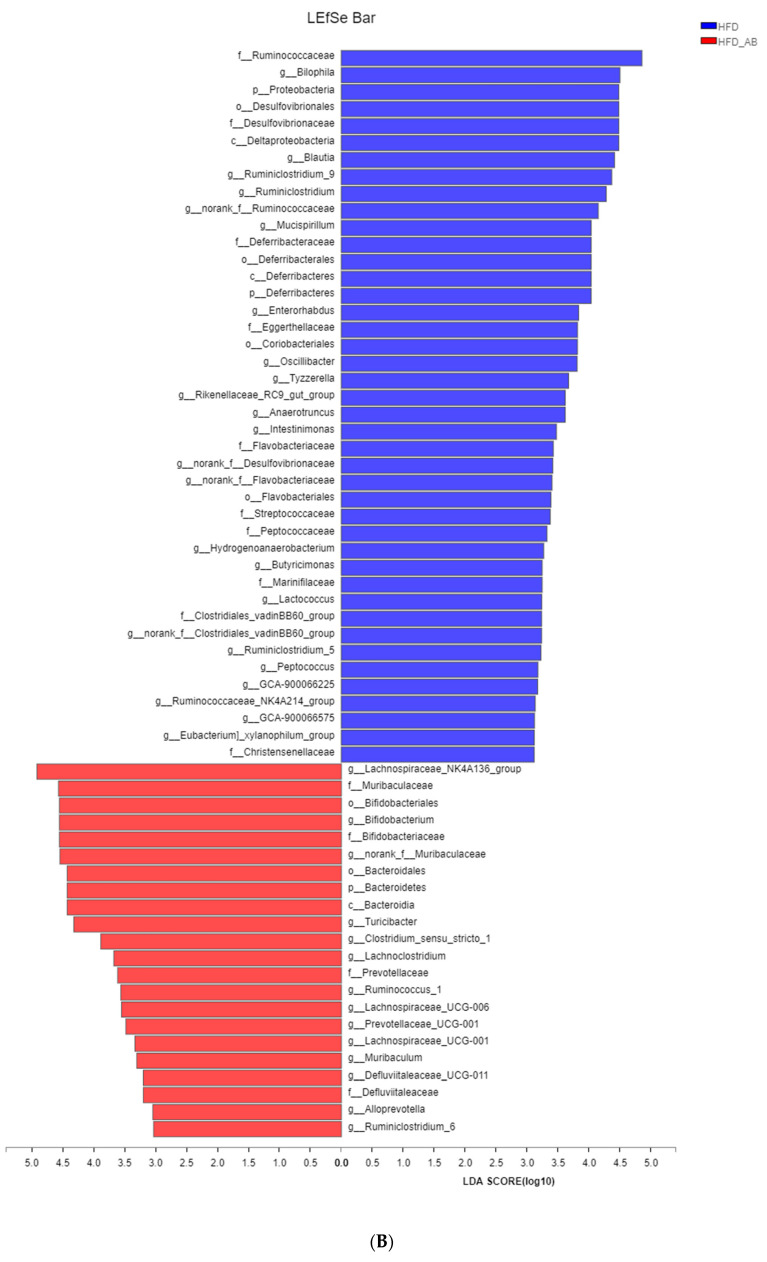
Key phylotypes of gut microbiota. (**A**) Cladogram generated from LEfSe analysis showing the relationship among taxons, and (**B**) linear discriminant analysis (LDA > 3) scores derived from LEfSe analysis.

Gut microbiota affects metabolic function and is important in the development of obesity. α diversity reflects the richness and diversity of a microbial community. Community richness refers to the number of species. Community diversity is a comprehensive index of species richness and evenness. The ACE and Chao indices represent the community richness and are often used to estimate the total number of species. Simpson and Shannon indices represent the community diversity. The greater the Shannon value, the higher the community diversity, while the Simpson index is the opposite [16]. Compared with the NCD group, HFD feeding led to significantly decreased community richness (Figure 5A,B), and a significant increase in the community diversity (Figure 5C,D). Conversely, adzuki bean supplementation showed significantly increased community richness, but no significant change in community diversity. By using β diversity analysis based on PCoA and NMDS, the three groups formed clusters separated from each other, indicating that gut microbiota composition changed significantly in response to adzuki bean supplementation and HFD feeding (Figure 5E,F). At the phylum level, *Firmicutes*, *Bacteroidetes*, *Actinobacteria*, *Verrucomicrobia*, and *Proteobacteria* were abundant (Figure 6A). Compared with the NCD group, HFD feeding significantly increased the ratio of *F*/*B*, but this effect was reversed by adzuki bean supplementation (Figure 6B). At the genus level, HFD feeding significantly decreased the abundance of *Bifidobacterium* and *norank_f_Muribaculaceae*, and enriched *Bilophila*, *Blautia*, *Ruminiclostridium_9*, and *Ruminiclostridium* compared with the NCD group (Figure 6C). Adzuki bean supplementation not only reversed these effects, but also significantly enriched *Lachnospiraceae_NK4A136_group*. The cladogram corresponding to five phylogenetic levels (from phylum to genus) generated from LEfSe analysis indicates that different dietary interventions led to specific changes in the bacterial taxa (Figure 7A). Further, the LDA scores from LEfSe analysis show that HFD-fed mice were rich in *Bilophila*, *Blautia*, *Ruminiclostridium*, *Ruminiclostridium_9*, and *Desulfovibrionaceae* compared with the NCD group (Figure 7B). Adzuki bean supplementation not only reversed these effects, but also significantly enriched *Lachnospiraceae_NK4A136_group*, *norank_f_Muribaculaceae*, *Bifidobacterium*, *Turicibacter*, *Lachnoclostridium*, *Prevotellaceae*, *Ruminococcus_1*, *Muribaculum*, and *Alloprevotella* (Figure 7B).

### 3.6. Gut Microbial Metabolic Functions

**Figure 8:**
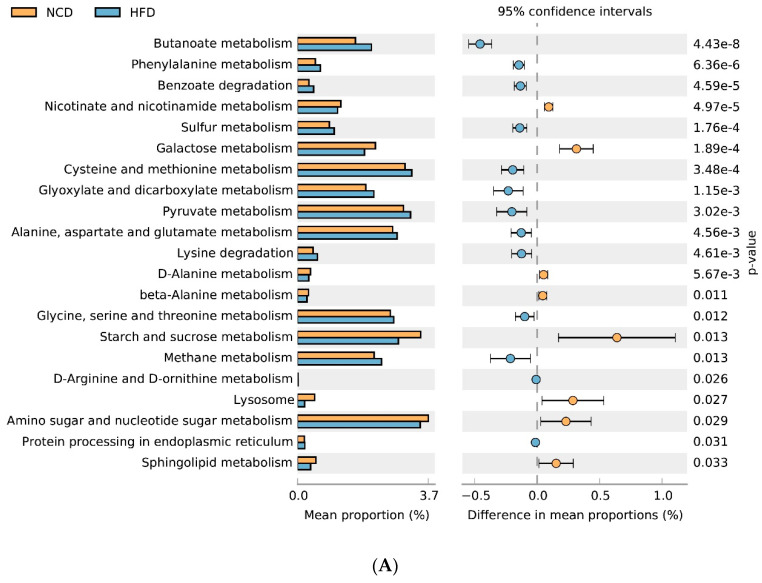

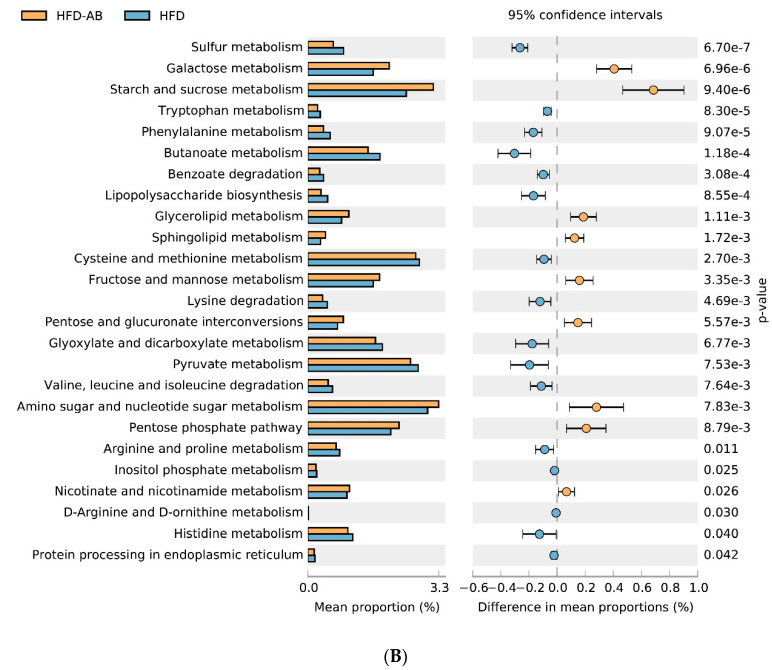
Predicted metabolic profile of the fecal microbiome after different treatments. (**A**) HFD vs. NCD; (**B**) HFD-AB vs. HFD. 16S rRNA data were analyzed as indicated by the PICRUSt2. Statistical significance difference among treatment groups based on Welch’s *t*-test (*p* < 0.05) in STAMP. The colored circles represent 95% confidence intervals calculated using Welch’s inverted method.

The 16S rRNA data were mapped to the KEGG pathway, and the metabolic characteristics of gut microbiota under different dietary interventions were predicted based on the abundance distribution of each metabolic pathway in each sample. Compared with the NCD group, HFD feeding significantly reduced the metabolism of sphingolipids, starch and sucrose, amino sugar and nucleotide sugars, galactose, nicotinate, and nicotinamide; and enhanced sulfur metabolism and cysteine and methionine metabolism (Figure 8A). Adzuki bean supplementation not only reversed the effects of HFD feeding, but it also reduced lipopolysaccharide biosynthesis and valine, leucine, and isoleucine degradation. It also enhanced the pentose phosphate pathway, glycerolipid metabolism, pentose and glucuronate interconversions, and fructose and mannose metabolism (Figure 8B).

## 4. Discussion

Our previous study showed that type 2 diabetic mice fed a HFD supplemented with 30% adzuki bean for 8 weeks had significantly decreased glycosylated serum protein, fasting blood glucose, LDL-C, and TC, and improved glucose tolerance [15]. The Dietary Guidelines for Chinese Residents (2016) suggested a 30% maximum intake level for pulses. In the current study, health benefits were also observed in mice with obesity induced by HFD when the adzuki bean supplementation level was as low as 15%. Supplementation with 15% adzuki bean significantly reversed HFD-induced obesity, regulated lipid metabolism disorders, reduced obesity-related liver function damage and hepatic steatosis, improved glucose homeostasis, and alleviated metabolic endotoxemia. Importantly, the effects of adzuki bean supplementation on improving obesity and related complications induced by HFD was not related to reduced energy consumption (Figure 1F). In addition, adzuki bean supplementation regulated gut microbiota dysbiosis and promoted the proliferation of beneficial bacteria.

HFDs can disrupt the balance between lipid absorption and metabolism, resulting in lipid metabolism disorders. In the present study, adzuki bean supplementation significantly alleviated HFD-induced lipid accumulation and weight gain, and reduced serum TG, TC, and LDL-C levels, and thereby improved lipid metabolism disorders in HFD-fed mice (Figure 1A–E and Figure 2A). Both flavonoids and saponins of adzuki bean can significantly alleviate adipose tissue accumulation and reduce serum levels of TG, TC, and LDL-C in obese mice [10]. A previous study has also shown that the hydrolyzed peptide of adzuki bean protein has cholesterol-lowering activity [12]. The liver is a critical organ for lipid and carbohydrate metabolism. Abnormal lipid metabolism results in liver lipid accumulation [17]. The activity of ALT and AST are the most sensitive indicators of liver function and when elevated may indicate the presence of inflammation, toxicity, and tissue trauma [18]. The present study showed that adzuki bean supplementation significantly inhibited AST and ALT activity and reduced hepatic steatosis caused by HFD, indicating a protective effect of adzuki bean on the liver (Figure 2B and Figure 3A,B). Whole adzuki bean and hot water extracts from adzuki bean can attenuate nonalcoholic fatty liver disease and HFD-induced hepatic steatosis, respectively [13,19]. Obesity-related metabolic syndrome is usually associated with changes in glucose tolerance [20]. Adzuki bean treatment significantly reduced HFD-induced high levels of serum insulin and glucose, and the AUC of the OGTT test (Figure 4A–D). Adzuki bean polysaccharides and extruded adzuki bean protein have also been reported to have hypoglycemic effects [21,22].

Gut microbiota is important in maintaining homeostasis and metabolic disorders, and is closely related to obesity [23]. The gut microbiota composition can be reshaped through the interaction between intestinal microorganisms and dietary components [24]. More and more evidence shows that HFD leads to a decline in community richness [25], which may be due to the lack of healthy microflora induced by HFD [26]. In the current study, HFD feeding led to significantly decreased community richness, which was reversed by adzuki bean supplementation (Figure 5A,B). Analysis of β diversity also showed that the gut microbiota composition changed significantly in response to adzuki bean supplementation and HFD feeding (Figure 5E,F), which was consistent with previous studies [19]. The *F*/*B* ratio is a biomarker of gut microbiota imbalances and is closely associated with obesity [27]. Dietary supplementation with adzuki bean paste can reduce the *F*/*B* ratio [6]. Furthermore, the intake of polyphenols and dietary fiber promotes a beneficial *F*/*B* ratio [28,29], and adzuki bean is rich in polyphenols and dietary fiber (Supplementary Table S1). In contrast, an increasing *F*/*B* ratio induced by HFD leads to enhanced energy collection and inflammation [30]. Importantly, lean individuals have a lower *F*/*B* ratio than obese individuals [31]. In the present study, compared with the HFD group, adzuki bean supplementation significantly decreased the *F*/*B* ratio (Figure 6B), which may contribute to relieving weight gain (Figure 1A–C). Therefore, adzuki bean supplementation significantly reversed gut microbiota dysbiosis induced by HFD and returned it to a level similar to normal based on analysis of α and β diversity and the *F*/*B* ratio.

Legume consumption promotes the proliferation of *Bifidobacterium*, which is beneficial in the host [32]. Polyphenols are beneficial to the growth, proliferation, and survival of gut microbiota [33]. Many studies have shown that polyphenols significantly enrich *Bifidobacterium* [34]. Certain non-digestible carbohydrates, such as dietary fiber, are potential substrates for the fermentation of *Bifidobacterium*, and leguminous diets rich in dietary fiber can promote the proliferation of *Bifidobacterium* [35]. Undigested protein may also enter the large intestine and undergo bacterial metabolism [36]. Many studies have reported that dietary protein regulates the gut microbiota composition and promotes the growth of *Bifidobacterium* [37,38]. Adzuki bean is rich in polyphenols, dietary fiber, and protein (Supplementary Table S1), which may contribute to the enrichment of *Bifidobacterium*. Importantly, *Bifidobacterium* supplementation has been reported to significantly reduce plasma TG level in obese mice [39]. In addition, *Bifidobacterium* can alleviate obesity, regulate glucose homeostasis, and produce short-chain fatty acids (SCFAs) [40,41]. SCFAs can affect glucagon like peptide 1, promote the cell differentiation of adipose tissue, and reduce the production of liver fat, thereby alleviating lipid accumulation and regulating glycolipid metabolism [42]. In the present study, compared with the HFD group, adzuki bean supplementation significantly increased the abundance of *Bifidobacterium*, which may contribute to reducing serum TG level, alleviating obesity, regulating glucose homeostasis, and mitigating hepatic steatosis (Figure 1A–C, Figure 2A, Figure 3A,B and Figure 4A–D). 

The loss of beneficial bacteria and the spread of harmful bacteria are the pathogenic factors of metabolic diseases [43]. *Muribaculum*, *Turicibacter*, and *Lachnospiraceae_NK4A136_group* are related to the regulation of body weight [44,45,46]. In addition, *Turicibacter* and *Lachnospiraceae_NK4A136_group* are also associated with improving insulin sensitivity [45,46]. SCFA-producing bacteria (*Lachnoclostridium*, *Ruminococcus_1*, *Alloprevotella*, and *norank_f_Muribaculaceae*) can not only cause lipolysis and fatty acid oxidation, improve insulin sensitivity, and alleviate host obesity [47,48], but also inhibit liver cholesterol synthesis [49]. In contrast, *Ruminiclostridium_9* can cause abnormal lipid regulation [50]. *Blautia* is related to the development of glucose metabolism disorders [51]. We found that adzuki bean supplementation significantly relieved obesity, lipid metabolism disorders, insulin resistance, and hepatic steatosis induced by HFD (Figure 1A–C, Figure 2A, Figure 3A,B and Figure 4A–D). The beneficial effects of adzuki bean supplementation may be due to enriched *Lachnoclostridium*, *Ruminococcus_1*, *norank_f_Muribaculaceae*, *Alloprevotella*, *Muribaculum*, *Lachnospiraceae_NK4A136_group*, and *Turicibacter*, and depleted *Blautia* and *Ruminiclostridium_9*.

LPS, the main component of outer membrane of Gram-negative bacteria, is believed to be enriched by gut microbiota [52]. Serum LPS content can reflect the change of intestinal permeability, and then reflect the level of gut microbiota disorders [53]. HFD feeding can promote the proliferation of endotoxin-producing bacteria [54]. Meanwhile, HFDs can increase the permeability of the intestinal mucosal barrier, causing more LPS to enter the circulatory system and promoting systemic inflammation and obesity [55]. *Desulfovibrionaceae* and *Bilophila* promote the production of LPS [56,57]. In contrast, *Bifidobacterium* is negatively correlated with LPS levels in obese individuals [40,41]. In the present study, adzuki bean supplementation significantly decreased the abundance of *Desulfovibrionaceae* and *Bilophila*, and enriched *Bifidobacterium,* which may contribute to reducing the level of serum LPS and alleviating metabolic endotoxemia (Figure 2C).

Many studies have shown that legume consumption, such as mung bean, can promote intestinal and host health by decreasing the abundance of HFD-dependent taxa [32]. The intestinal barrier is the body’s first line of defense against the invasion of pathogens. *Desulfovibrionaceae* promotes the degradation of intestinal mucus [58]. *Ruminiclostridium* is involved in intestinal dysfunction [59]. In contrast, acetic acid produced by the fermentation of *Bifidobacterium* can enhance the intestinal barrier and promote intestinal peristalsis, so as to inhibit the colonization of pathogenic bacteria and reduce the intake of toxins [60]. *Prevotellaceae* can reduce endothelial permeability, prevent the host from being exposed to endotoxins, and promote the improvement of intestinal function [61]. *Ruminococcus_1*, which promotes the production of SCFAs, is beneficial to the growth of epithelial cells [62]. Increasing levels of SCFAs reduce the pH value of the colon, thereby preventing the overgrowth of pathogens, which is conducive to intestinal health [63]. In the present study, adzuki bean supplementation may reduce the abundance of *Desulfovibrionaceae* and *Ruminiclostridium*, and increase the abundance of *Bifidobacterium*, *Prevotellaceae*, and *Ruminococcus_1* to inhibit the colonization of pathogenic bacteria, prevent intestinal harmful substances from entering the circulatory system, and maintain intestinal and host health, which was supported by the significantly reduced serum LPS level in HFD-AB group mice (Figure 2C).

Gut microbiota can change host metabolism and have metabolic function and activity [64]. Adzuki bean supplementation alleviated HFD-induced disorders of lipid metabolism (glycerolipid and sphingolipid metabolism) and carbohydrate metabolism (amino sugar and nucleotide sugar metabolism, galactose metabolism, starch and sucrose metabolism, pentose and glucuronate interconversions, fructose and mannose metabolism, and pentose phosphate pathway), which was consistent with significantly reduced serum lipid levels and improved glucose homeostasis in HFD-AB group mice (Figure 2A and Figure 4A–D). Branched-chain amino acids include leucine, valine, and isoleucine. An intermediate product of valine metabolism, 3-hydroxyisobutyrate, can increase lipid accumulation and induce insulin resistance [65]. The inhibited valine, leucine, and isoleucine degradation may contribute to alleviating HFD-induced lipid accumulation and insulin resistance by adzuki bean supplementation (Figure 1D,E and Figure 4A–D). The HFD group has a higher relative abundance of lipopolysaccharide biosynthesis, which was consistent with the high serum LPS levels, but adzuki bean treatment has a significant negative regulatory effect on the content of LPS (Figure 2C). Furthermore, cysteine and methionine metabolism is part of sulfur metabolism, which promotes the production of H_2_S [66]. H_2_S has adverse effects on the energy metabolism of the colonic epithelium, which promotes harmful intestinal substances from entering the circulatory system [67]. Compared with the HFD group, the lower level of serum LPS in the HFD-AB group also supported inhibited cysteine and methionine metabolism (Figure 2C).

Beans are rich in protein, dietary fiber, polysaccharides, and phytochemicals [68]. The hydrolyzed peptide of adzuki bean protein has cholesterol-lowering activity [12]. The seed coat of beans is rich in dietary fiber [69], and their metabolites can promote the proliferation of beneficial bacteria by regulating gut microbiota dysbiosis [70]. Adzuki bean dietary fiber can alleviate visceral fat accumulation, reduce the levels of serum lipid, and modulate the composition of gut microbiota in rats fed a normal diet [6]. Polysaccharides are one of the main biologically active substances of beans. Adzuki bean polysaccharides are reported to significantly reverse insulin resistance and dyslipidemia in diabetic rats [11]. In addition, saponins from adzuki bean and extracts containing adzuki bean polyphenols can significantly regulate lipid metabolism disorders in obese mice [10,19]. Due to the complex composition of whole adzuki bean, identifying the specific substances of adzuki bean responsible for alleviating obesity and regulating gut microbiota dysbiosis in HFD-fed mice has yet to be done.

In conclusion, adzuki bean supplementation can significantly alleviate HFD-induced obesity, regulate lipid metabolism disorders, reduce liver function damage and hepatic steatosis associated with obesity, and improve glucose homeostasis and metabolic endotoxemia. In addition, the health benefits of adzuki bean may be associated with regulating gut microbiota dysbiosis, inhibiting the spread of harmful bacteria, and promoting the proliferation of beneficial bacteria. These results suggest that adzuki bean can be regarded as a functional food to prevent and treat obesity and related complications. In the future, more work is needed to verify the anti-obesity mechanism of adzuki bean supplementation and identify the specific bioactive components involved.

## Data Availability

All data that support the findings of this study are available from the corresponding author on reasonable request.

## Supplementary Materials

The following are available online at https://www.mdpi.com/article/10.3390/nu13093240/s1, Table S1: Composition of adzuki bean (g/100 g), Table S2: Composition of experimental diets.
