# Supplementary material for: Adzuki Bean Alleviates Obesity and Insulin Resistance Induced by a High-Fat Diet and Modulates Gut Microbiota in Mice
Source: Nutrients. 2021 Sep 17;13(9):3240. doi: 10.3390/nu13093240 (PMC8466346; doi:10.3390/nu13093240)
Supplement: Supplementary file 1 [file nutrients-13-03240-s001.zip › nutrients-1375977-supplementary.pdf]

Supplementary Table S1. Composition of adzuki bean (g/100g).

Basic nutritional composition	
Moisture	12.19±0.08
Protein	22.06±0.18
Fat	1.39±0.01
Ash	3.031±0.004
Sucrose	0.523±0.002
**Bioactive substances**	
Total polyphenols	0.6281±0.0015
Total catechins	0.1715±0.0015
Epicatechin/EC	0.1467±0.0013
Epigallocatechin/EGC	0.0249±0.0008
Epigallocatechin gallate/EGCG	0.0069±0.0001
Catechin/C	0.0073±0.0003
Epicatechin gallate/ECG	0.0017±0.0000
Total dietary fiber	9.17±0.11
Total saponins	0.2897±0.0105

Data are expressed as mean ± standard deviation (n = 3).

Supplementary Table S2. Composition of experimental diets

Ingredient (g/kg)	NCD	HFD	HFD-AB
Adzuki bean	0.00	0.00	150.00
Casein, 80 Mesh	189.56	258.45	220.77
L-Cystine	2.84	3.88	3.88
Corn Starch	479.79	0.00	0.00
Maltodextrin 10	118.48	161.53	58.68
Sucrose	65.21	88.91	88.41
Cellulose, BW200	47.39	64.61	59.44
Soybean Oil	23.70	32.31	28.31
Lard	18.96	316.60	316.60
Mineral Mix	9.48	12.92	12.92
Dicalcium Phosphate	12.32	16.80	16.80
Calcium Carbonate	5.21	7.11	7.11
Potassium Citrate, 1 H_2O	15.64	21.32	21.32
Vitamin Mix, V10001	9.48	12.92	12.92
Choline Bitartrate	1.90	2.58	2.58
FD&C Yellow Dye #5	0.04	0.00	0.00
FD&C Blue Dye #1	0.01	0.06	0.00
Total	1000	1000	1000
% Energy and source			
Protein	20	20	20
Carbohydrate	70	20	20
Fat	10	60	60
Total	100	100	100

NCD, normal control diet; HFD, high-fat diet; HFD-AB, high-fat diet supplemented with adzuki bean.
